# Efficacy of low-fat milk and yogurt fortified with vitamin D_3_ on systemic inflammation in adults with abdominal obesity

**DOI:** 10.1186/s41043-022-00283-0

**Published:** 2022-03-02

**Authors:** Payam Sharifan, Mohammad Rashidmayvan, Zahra Khorasanchi, Susan Darroudi, Azam Heidari, Fatemeh Hoseinpoor, Hassan Vatanparast, Mohamad Safarian, Saeid Eslami, Asma Afshari, Zahra Asadi, Hamideh Ghazizadeh, Mohammad Bagherniya, Hamed Khedmatgozar, Gordon Ferns, Mitra Rezaie, Majid Ghayour Mobarhan

**Affiliations:** 1 Student Research Committee, School of Medicine, Mashhad University of Medical Sciences, Mashhad, Iran; 2 Department of Nutrition, School of Medicine, Mashhad University of Medical Sciences, Mashhad, Iran; 3 International UNESCO Center for Health Related Basic Sciences and Human Nutrition, Mashhad University of Medical Sciences, 99199-91766 Mashhad, Iran; 4 Department of Nutrition Sciences, Varastegan Institute for Medical Sciences, Mashhad, Iran; 5 College of Pharmacy and Nutrition, University of Saskatchewan, Saskatoon, Canada; 6 Department of Medical Informatics, School of Medicine, Mashhad University of Medical Sciences, Mashhad, Iran; 7 Department of Community Nutrition, School of Nutrition and Food Science, Food Security Research Center, Isfahan University of Medical Sciences, Isfahan, Iran; 8 Center for Biotechnology and Genomics, Texas Tech University, Lubbock, TX USA; 9 Division of Medical Education, Brighton and Sussex Medical School, Brighton, UK

**Keywords:** Fortification, Inflammation, Obesity, Vitamin D, Low-fat dairy

## Abstract

**Background:**

The prevalence of vitamin D deficiency is increasing globally and is associated with an increased risk of metabolic syndrome, autoimmune disease, and cardiovascular disease. Vit D deficiency is also associated with increased systemic inflammation. The current study aimed to determine the efficacy of low-fat milk and yogurt fortified with 1500 IU nano-encapsulated vitamin D, on systemic inflammation in abdominal obese participants.

**Method:**

This multi-center study was conducted using a 2.5-month parallel total-blind randomized clinical trial design. Two hundred and eighty nine subjects were allocated to four groups: low-fat milk fortified by 1500 IU nano-encapsulated vitamin D_3_ (200 mL/day). Simple milk (200 mL/day), low-fat yogurt fortified by 1500 IU nano-encapsulated vitamin D_3_ (150 g/day), and simple yogurt (150 g/day).

**Results:**

The results showed that serum levels of neutrophils, lymphocytes, platelets and red blood cell distribution width (RDW) were significantly lower before and after the intervention in fortified dairy groups. The results showed that serum levels of neutrophils, lymphocytes, platelets, and RDW before and after intervention in the fortified dairy groups were significantly lower (*p* < 0.05). The values of = neutrophil to lymphocyte ratio (NLR), platelets to lymphocyte ratio, and RDW to platelets ratio (RPR) reduced significantly in the fortification group (*p* < 0.05).

**Conclusion:**

Fortification with nano-encapsulated vitamin D_3_ of dairy products may decrease inflammation in individuals with abdominal obesity.

## Introduction

Inflammation plays an important role in the progress of chronic diseases such as obesity, cardiovascular disease, type 2 diabetes mellitus and cancer [1]. Obesity is considered a significant health care problem and is a complex multifactorial condition. The prevalence of overweight and obesity has increased worldwide in the past five decades, and approximately a third of the world's population is now classified as overweight or obese. Of great concern is abdominal obesity, one of the most common health issues with increasing incidence in middle-aged people globally [2]. In the last studies, the prevalence of abdominal obesity in Iran was described to be 52.8% and 44.4% in men and women, respectively [3]. Several studies have shown that abdominal obesity may be associated with low-grade of systemic inflammation, which is characterized by an increase in pro-inflammatory cytokines, hematological inflammatory parameters and acute phase proteins such as C-reactive protein (CRP) [4–6]. The neutrophil to lymphocytes ratio (NLR) is an important inflammatory index affected by health status and lifestyle [7]. Also, recent animal and human experimental studies have shown that increased WBC and neutrophils counts are potentially related to obesity and are obesity correlated inflammatory markers [8, 9]. Red blood cell distribution width (RDW) is a parameter routinely measured by advanced hematology analyzers. The RDW, an indicator of a routine blood test, shows the average volume and variety of red blood cells and is usually combined with other clinical indicators to diagnose anemia [10]. RDW may be a new inflammatory biomarker [11], and a high RDW is reported to be associated with metabolic syndrome (MetS) [12]. Further evidence for the potential connection between prognosis and RDW is chronic inflammation and even low-intensity inflammation might play a significant role in atherogenesis and platelet activation [11].

A low plasma of 25-hydroxy-vitamin D is commonly seen in obesity. Vitamin D may be involved in etiology of obesity, and its complications, such as hypertension, insulin resistance, and low-grade inflammation [13]. Many clinical studies have proved that vitamin D deficiency might increase inflammation and immune activation [14]. Moreover, researches indicate that the adaptive immune response is regulated by vitamin D in various autoimmune and inflammatory diseases [15]. One study reported that patients with vitamin D deficiency showed increased serum high sensitivity C-reactive protein (hs-CRP) levels [16].

Akbas et al. have reported that patients with low serum 25(OH)D levels have higher NLR [17]. In a 9-week RCT on patients receiving 50,000 IU/week, Tabatabaeizadeh et al. examined how 25(OH)D supplementation may affect inflammatory markers. They have demonstrated that a significant increase in serum 25(OH)D resulted in an reduction in serum hs-CRP, and NLR [18]. The concentration of vitamin D3 in the bone marrow is more than two hundred times higher than in the blood, and lack of vitamin D3 cause cell proliferation and erythropoiesis, and a slight drop in levels of serum vitamin D3 cause dysfunction of red blood cells in the bone [19, 20]. These results indicate the potential advantageous impacts of vitamin D supplementation in reducing the risk and adverse events of inflammatory diseases; however, the precise effect remains illuminated in large studies.

Although few studies have shown the consequence of vitamin D supplementation on inflammatory markers, the lack of data regarding the efficacy of vitamin D fortification in dairy products remains unclear. In this study, we aimed to determine the effectiveness of low-fat milk and yogurt fortified by 1500 IU nano-encapsulated vitamin D on serum levels of C-reactive protein, WBC, neutrophil, lymphocyte, platelets, RDW, NLR, PLR and RPR in 2/5 months trial.

## Methods

### Study design

The study was designed as a randomized, total-blind clinical trial, using fortified low-fat dairy products containing nano-encapsulated vitamin D (1500 IU), and was conducted for 2.5-month in Mashhad, Iran between January 2019 and March 2019. Before the study, the protocol was approved by the Ethics Committee of the National Institute for Medical Research Development (NIMAD; protocol ID: IR.NIMAD.REC.1396.027).

This report was a pilot study, as a part of *Survey of ultraviolet intake by nutritional approach* (SUVNIA) study (Trial registration: IRCT20101130005280N27, wwww.IRCT.ir). This trial was to evaluate the effectiveness of 1500 IU vitamin D_3_ on physical and mental aspects of health in abdominal obese adults as a clinical trial.

### Sample size

The sample size was determined as pilot using an 80 percent power and a 0.5 effect size. The sample size was determined to be 255 people. With a 10% dropout rate, the final sample size was calculated to be at least 280 participants (70 in each group) [22].

### Participants

We recruited middle-aged adults (30–50 years) with abdominal obesity (*n* = 306). Among recruited subjects, 289 subjects finished the trial. Abdominal obesity was considered according to the International Diabetes Federation (IDF) as waist circumference (WC) ≥ 94 cm for men and ≥ 80 cm for women [21]. All participants complete general information questionnaires, including demographic information, history of any diseases, and current treatments before starting the trial. The intervention will be considered for 2.5 months. In each intervention group, one of the products (milk or yogurt) was fortified with vitamin D_3_, and each control group received one non-fortified dairy product (milk or yogurt). We used similar packaging and same taste and smell of dairy products in both intervention and control groups. Only the intervention groups consumed dairy products containing 1500 international units of nano-encapsulated vitamin D_3_.

### Inclusion and exclusion criteria

Inclusion criteria included participants aged 30 to 50 years, providing written, informed consent for participation in the study. Exclusion criteria included previously taking any vitamin d supplements three months before the study; not having chronic liver disease, cystic fibrosis, Crohn's. Other exclusion criteria included: an intention to lose weight, women who were pregnant or lactating, those with a history of lactose intolerance or sensitivity, using supplements containing vitamin D or any medications with interaction with vitamin D (corticosteroids, anticonvulsants, antidepressant, sleeping medications) in the three months before the trial.

### Outcome measurements

The study’s primary outcome was a change in levels of inflammatory factors after 2.5 months of intervention in people with abdominal obesity. Serum levels of serum high sensitivity C-Reactive Protein (hs-CRP), white blood cells, lymphocytes, neutrophils, platelets, and RDW were assessed.

The secondary outcome of the study was a change in the ratios that can predict inflammation in these participants after the 2.5-month trial period. These ratios complement other inflammatory factors. The ratio of neutrophils to lymphocytes is the ratio of platelets to lymphocytes and the ratio of RDW to platelets. The variables evaluated in the study are routine and inexpensive, and together they were reported to predict systemic inflammation in patients and be assessed in many diseases.

### Randomization and blinding

Stratified block allocation was done for eligible subjects for center and sex status with ratio 1:1:1:1 to receive fortified low fat milk containing 1500 IU nano-encapsulated vitamin D_3_/per serving (200 mL/day), simple low-fat milk (200 mL/day), fortified low fat yogurt containing 1500 IU nano-encapsulated vitamin D_3_/per serve (150 g/day) and simple low-fat yogurt (150 g/day) for ten weeks' trial. Closed envelopes containing A or B labels were used for the placebo groups and interventions, respectively. Envelopes were opened in order in front of each participant. The list of allocations by the faculty of Medicine was safe and there was no access to researchers until the end of the study. Blinding was implemented for subjects, investigators, statisticians, and staff who allocated subjects into the groups (total blinding).

### Nano-encapsulated formulation and dairy products manufacture

Ingredients used for generating nanocapsules were: precirol a solid lipid, oleic acid as liquid lipid, vitamin D as the bioactive fatty core, ploxamer 188 as a surfactant and deionized water. All components were homogenized with high tensile stress using ultrasound.

Fortification of low-fat milk and yogurt was undertaken at the Salamat dairy factory under the supervision of the faculty of Food Science and Technology. (Ferdowsi University of Mashhad, Iran). Nutritional information for each 100 g milk and yogurt included: 56 kcal, sugar-free, protein 7 g fat 3 g, and trans fatty acids 0.04 g. The delivery and consumption of products (intervention or placebo) were done on the day of production, or the next day.

### Laboratory measurements

Before and after intervention we obtained 4 mL of venous blood from each participant. Samples were collected and centrifuged (3000 RPM, and 15 min), they were stored at -70 °C by a trained technician. Hematologic factors including white blood cell count (WBC), Neutrophil, Lymphocyte, Neutrophil to lymphocyte ratio and platelets to lymphocyte ratio were assessed by cell counter machine. Serum high sensitivity C-reactive protein (hs-CRP) was assessed by a polyethylene glycol (PEG)—enhanced immunoturbidimetry method (mg/L) using an Alcyon analyzer device (Abbott, Chicago, IL, USA).

### Statistical analysis

All statistical analyses performed with the SPSS version 22.0. We used Kolmogorov–Smirnov test to check the normal distribution of all variables. We used independent t-test and ANOVA to check the analogy of quantitative variables in two groups, and determine the differences before and after study. ANOVA test was followed by Bonferroni correction. And we applied a non-parametric equation for non-normal variables.

## Results

All 280 participants of the study were assessed, and the demographic information are shown in Table 1. Participants were followed for 2.5 months after providing informed consent, and divided into two groups: low-fat milk fortified with vitamin D and low-fat yogurt fortified with vitamin D. Baseline characteristics of the study participants are recorded in Table 1. There were no significant differences in baseline features and biochemical characteristics between the intervention group (*n* = 140) and the control group (*n* = 140).

**Table 1:** Clinical and biological characteristics of the participants at baseline

	Total		Milk		Yogurt	
	Intervention	Control	Intervention	Control	Intervention	Control
Age (y)	41.9 ± 7.77	41.75 ± 7.88	40.42 ± 8.03	40.26 ± 8.25	43.47 ± 7.21	43.19 ± 7.25
*p*-value*	0.87		0.9		0.82	
*Sex*						
Male	64 (47.8%)	68 (48.6%)	34 (49.3%)	36 (52.2%)	30 (46.2%)	32 (45.1%)
Female	70 (52.2%)	72 (51.4%)	35 (50.7%)	33 (47.8%)	35 (53.8%)	39 (54.9%)
*p*-value*	0.49		0.43		0.51	
Serum 25(OH)D (ng/mL)	14.75 ± 5.18	14.97 ± 5.07	14.71 ± 5.54	13.66 ± 5.68	14.8 ± 4.8	15.47 ± 3.92
*p*-value*	0.72		0.35		0.055	

*Data are expressed as mean ± standard deviation and were tested by ANOVA *t* test

As shown in Table 2, the results demonstrate a significant decrease in serum levels of neutrophils, lymphocytes, platelets, and RDW before and after the intervention in the low-fat milk fortified with vitamin D group. The values of NLR, PLR, and RPR show a significant decrease in the study group. In Table 3, which is related to the vitamin D-fortified yogurt group, the results show that blood levels of neutrophils, lymphocytes, platelets, and RDWs were significantly lower than before the intervention. Also, PLR and RPR values in the study group had a significant decrease compared to before the intervention. NLR changes between the study group and control showed a statistically significant reduction.

**Table 2:** Effect of intervention on inflammation markers according to milk consumption

	Before intervention	After intervention	*p*-value*	Changes	*p*-value*
*Serum 25(OH)D (ng/mL)*					
Intervention	14.08 ± 5.15	19.1 ± 5.69	< 0.001	5.02	< 0.001
Control	14.1 ± 5.04	13.89 ± 5.85	0.71	0.21	
*hs-CRP (mg/L)*					
Intervention	1.1 (0–3.25)	1.15 (0.4–2.92)	0.16	− 0.25	0.77
Control	1.15 (0.1–3.75)	1.4 (0.5–4.2)	0.75	0.25	
*WBC (billion cells/L)*					
Intervention	6.56 ± 1.57	6.35 ± 1.68	0.099	− 0.05	0.37
Control	6.64 ± 1.5	6.57 ± 1.35	0.64	0.07	
*Neutrophil (percent)*				7.37	
Intervention	61.75 ± 6.57	54.38 ± 5.44	< 0.001	1.06	0.5
Control	58.8 ± 6.97	57.74 ± 6.22	0.12		
*Lymphocyte (percent)*				0.45	
Intervention	35.97 ± 7.2	35.52 ± 7.19	0.5	− 0.29	0.75
Control	35.45 ± 6.97	35.74 ± 6.42	0.58		
*Platelets (billion/L)*					
Intervention	267.96 ± 35.01	214.82 ± 36.77	< 0.001	53.14	0.49
Control	251.88 ± 56.33	236.42 ± 47.96	0.001	15.46	
*RDW (percent)*					
Intervention	13.24 ± 0.78	11.61 ± 0.97	< 0.001	1.63	0.089
Control	13.12 ± 0.83	13.29 ± 1.03	0.066	− 0.17	
*NLR*					
Intervention	1.81 ± 0.51	1.62 ± 0.51	< 0.001	0.19	0.39
Control	1.76 ± 0.56	1.68 ± 0.45	0.1	0.08	
*PLR*					
Intervention	7.78 ± 2.12	6.35 ± 1.98	< 0.001	1.43	0.48
Control	7.44 ± 2.64	6.88 ± 2.25	0.004	0.56	
*RPR*					
Intervention	0.055 ± 0.1	0.05 ± 0.007	< 0.001	0.005	0.98
Control	0.058 ± 0.01	0.054 ± 0.013	0.003	0.004	

*Data are expressed as mean ± standard deviation and were tested by ANOVA test

**Table 3:** Effects of intervention on inflammation markers according to yogurt consumption

	Before intervention	After intervention	*p*-value*	Changes	*p*-value*
*Serum 25(OH)D (ng/mL)*					
Intervention	14.14 ± 5.04	20.88 ± 5.76	< 0.001	6.84	< 0.001
Control	17.34 ± 5.68	16.46 ± 3.93	0.1	0.88	
*hs-CRP (mg/L)*					
Intervention	1.25 (0.2–4.17)	1.1 (0.3–3.9)	0.005	0.15	0.8
Control	0.9 (0.05–3.2)	1.1 (0.32–4.52)	0.041	0.02	
*WBC (billion cells/L)*					
Intervention	6.56 ± 1.57	6.35 ± 1.68	0.099	0.21	0.3
Control	6.64 ± 1.5	6.57 ± 1.35	0.64	0.07	
*Neutrophil (percent)*					
Intervention	61.25 ± 6.01	55.45 ± 4.89	< 0.001	5.8	0.43
Control	58.38 ± 7.13	59.38 ± 4.63	0.29	1	
*Lymphocyte (percent)*					
Intervention	36.92 ± 6.52	34.17 ± 5.05	0.004	2.75	0.07
Control	36.18 ± 6.48	35.28 ± 4.45	0.058	0.9	
*Platelets (billion/L)*					
Intervention	273.81 ± 36.97	216.82 ± 44.23	< 0.001	56.99	0.108
Control	235.10 ± 53.07	230.44 ± 54.06	0.18	4.66	
*RDW (percent)*					
Intervention	13.18 ± 1.13	11.58 ± 1.47	< 0.001	1.6	0.39
Control	13.32 ± 0.93	13.35 ± 1.13	0.67	0.03	
*NLR*					
Intervention	1.73 ± 0.43	1.66 ± 0.33	0.3	0.07	0.14
Control	1.7 ± 0.52	1.77 ± 0.33	0.27	0.07	
*PLR*					
Intervention	7.66 ± 1.76	6.52 ± 1.8	< 0.001	1.14	0.74
Control	6.73 ± 2.08	6.79 ± 1.8	0.21	0.06	
*RPR*					
Intervention	0.056 ± 0.01	0.049 ± 0.008	< 0.001	0.07	0.101
Control	0.061 ± 0.02	0.06 ± 0.017	0.8	0.01	

*Data are expressed as mean ± standard deviation and were tested by ANOVA test

## Discussion

This study is the first parallel totally-blinded RCT investigating the efficacy of vitamin D-fortified milk and yogurt on markers of systemic inflammation; modifications in serum hs-CRP level and WBC, neutrophils, lymphocytes, platelets, RDW, Neutrophil-to-lymphocyte ratio (NLR), Platelets-to-lymphocyte ratio (PLR) and RDW-to-platelet ratio (RPR) distribution in among individuals with abdominal obesity. Our results showed that, compared with the control group, fortified low-fat dairy products containing 1500 IU nano-encapsulated vitamin D3/per serving in the intervention group resulted in a significant improvement in systemic inflammation.

Some studies defined the relationship between vitamin D and inflammation, although it remains controversial. Some researchers have suggested that inflammation diminishes vitamin D levels, while others claim that increasing vitamin D levels overcome inflammation [22, 23]. Vitamin D supplementation provoke lower parathyroid hormone production [24] because we know that lowering parathyroid hormone might cause to the annihilation of generation of inflammatory factors.

Yombi et al. have reported that the distribution of neutrophil-to-lymphocyte ratios (NLR) to indicate early inflammation could be compared with hs-CRP [25]. Previously, the inverse relationship of 25 (OH)D and hs-CRP concentration was determined [26]. In another study among 206 patients with inflammatory polyarthritis by increase of 10 ng/ml in the 25(OH)D concentration, the levels of hs-CRp have been reduced 26.2% [27]. Also, Shin et al. Examined the relationship between hs-CRP and neutrophils to lymphocytes ratio (NLR) with mortality in patients with acute myocardial infarction and found that high CRP and high NLR separately predict the all cause of death [28]. Both serum hs-CRP and NLR are markers of inflammation. NLR has been used to estimate inflammation in various patients, such as cardiovascular patients, chronic and malignant diseases [29–31]. Previous studies have revealed that deficiency of vitamin D is linked to dysfunction of endothelial due to inflammatory processes [32, 33]. Further, the association between NLR and atherosclerosis has already been illustrated [34].

Akbas et al. studied a retrospective study of 4120 patients with 25 (OH)D deficiency and showed a significant negative association between 25 (OH)D and CRP, NLR, and PLR values. They also reported that the easily calculated, practical, repeatable and cost-effective parameters of NLR and PLR could be used as indicators for endothelial inflammation and dysfunction [17]. Tabatabaizadeh et al. assessed hs-CRP and NRL levels before and after vitamin D supplementation in 580 adults and reported that hs-CRP and NLR levels decreased after supplementation. They stated that NLR could be used to track inflammation [18]. Mirchi et al. reported a significant relation of 25(OH)D with NLR in hemodialysis patients [35]. Another study showed higher NLR values in 25 patients with inadequate diabetes and emphasized an increased risk of cardiovascular disease in 25 (OH)D deficiency [36].

With respect to the association between count of WBC and vitamin D, Mellenthin et al. examined the connection of lack of vitamin D and WBC count in the healthy population of adults. They noticed insufficiency of vitamin D was related to WBC count in smokers (*n* = 718), compared to non-smokers (*n* = 2005) [37]. Yildirim et al. reported no significant difference in WBC count between vitamin D deficient patients and control groups [38]. NLR and WBC were also significantly negatively correlated with 25(OH) D in major depressive disorder subjects [39].

In the previous studies, both neutrophil and lymphocyte counts were increased, but these increases were in favor of neutrophils [40, 41]. Neutrophils and lymphocytes were increased with obesity degree and severity of metabolic syndrome by a similar quantity. Even NLR was increased with obesity degree and metabolic syndrome severity, but these increases were not significant. The association between RDW and Metabolic syndrome can be explained by increased inflammation, which shows a significant relationship between high RDW and C-reactive protein, white blood cells, and fibrinogen [42]. Proinflammatory cytokines suppress erythropoietin-induced erythrocyte maturation, which cause to increase in RDW [43, 44]. Previous studies have shown that structural changes exist in the erythrocyte membrane among women with features of metabolic syndrome [45].

A high RDW was significantly associated with older age and more co-morbidities (hypertension, diabetes mellitus, peripheral vascular disease, ischemic heart disease, myocardial infarction, and coronary artery bypass surgery [46]. RDW may be the prognosis of chronic inflammation, which believed that even low-intensity inflammation plays a key role in atherogenesis and may be responsible for platelet activation [47]. Lippi et al. illustrated the link between RDW and inflammatory markers, such as the erythrocyte sedimentation rate and high sensitivity CRP [6]. In previous studies, RDW was associated with the concentrations of interleukin-6, soluble tumor necrosis factors I and II receptor, and fibrinogen levels [6, 49, 50].

In addition, chronic inflammation leads to disorders of iron metabolism and reduces production as well as bone marrow response to erythropoietin, leading to impaired blood formation and increased RDW [48–50]. Another factor affecting RDW is vitamin D3 deficiency, which is a risk factor for CAD [51]. Vitamin D3 deficiency is responsible for cell proliferation and erythropoiesis, and the concentration of vitamin D3 in the bone marrow is more than two hundred times higher than in the blood. Even a slight decrease in serum vitamin D3 levels may lead to dysfunction of red blood cells in the bone [19, 51] but mega-dose vitamin D therapy did not have a significant effect on hematological parameters in children [52].

In this study, we found that 1500 IU vitamin D in the form of nano-encapsulated in low-fat dairy products significantly decrease serum levels of hs-CRP, WBC, neutrophil, lymphocyte, platelets, and RDW in participants with abdominal obesity. Also, NLR, PLR, and RPR ratios decreased significantly after the intervention. However, to the best of our knowledge, no study evaluates the effect of vitamin D-fortified dairy products as nano-encapsulated on systemic inflammatory in people with abdominal obesity. The available evidence suggests that vitamin D fortification can have a positive impact on systemic inflammation in abdominally obese individuals.

## Data Availability

The data that support the findings of this study are available from the corresponding author upon reasonable request.

## Abbreviations

NLR: Neutrophil to lymphocyte ratio
PLR: Platelets to lymphocyte ratio
RPR: RDW to platelets ratio
